# Assessment of Causal Direction Between Gut Microbiota and Inflammatory Bowel Disease: A Mendelian Randomization Analysis

**DOI:** 10.3389/fgene.2021.631061

**Published:** 2021-02-18

**Authors:** Zi-Jia Zhang, Hong-Lei Qu, Na Zhao, Jing Wang, Xiu-Yan Wang, Rong Hai, Bin Li

**Affiliations:** ^1^Department of General Surgery, Suzhou Ninth People’s Hospital, Suzhou, China; ^2^Inner Mongolia Medical University, Hohhot, China; ^3^Inner Mongolia Autonomous Region People’s Hospital, Hohhot, China; ^4^Suzhou Hospital of Anhui Medical University, Anhui, China; ^5^Inner Mongolia Autonomous Region Health Management Service Center, Hohhot, China

**Keywords:** mendelian randomization, gut microbiota, inflammatory bowel disease, ulcerative colitis, causal relationship

## Abstract

**Background:**

Recent studies have shown that the gut microbiota is closely related to the pathogenesis of Inflammatory Bowel Disease (IBD), but the causal nature is largely unknown. The purpose of this study was to assess the causal relationship between intestinal bacteria and IBD and to identify specific pathogenic bacterial taxa via the Mendelian randomization (MR) analysis.

**Materials and Methods:**

MR analysis was performed on genome-wide association study (GWAS) summary statistics of gut microbiota and IBD. Specifically, the TwinsUK microbiota GWAS (*N* = 1,126 twin pairs) was used as exposure. The UK inflammatory bowel disease (UKIBD) and the Understanding Social Program (USP) study GWAS (*N* = 48,328) was used as discovery outcome, and the British IBD study (*N* = 35,289) was used as replication outcome. SNPs associated with bacteria abundance at the suggestive significance level (α = 1.0 × 10^–5^) were used as instrumental variables. Bacteria were grouped into families and genera.

**Results:**

In the discovery sample, a total of 30 features were available for analysis, including 15 families and 15 genera. Three features were nominally significant, including one family (*Verrucomicrobiaceae*, 2 IVs, beta = −0.04, *p* = 0.05) and two genera (*Akkermansia*, 2 IVs, beta = 0.04, *p* = 0.05; *Dorea*, 2 IVs, beta = −0.07, *p* = 0.04). All of them were successfully replicated in the replication sample (*Verrucomicrobiaceae* and *Akkermansia P*_replication_ = 0.02, *Dorea P*_replication_ = 0.01) with consistent effect direction.

**Conclusion:**

We identified specific pathogenic bacteria features that were causally associated with the risk of IBD, thus offering new insights into the prevention and diagnosis of IBD.

## Introduction

Inflammatory bowel disease (IBD) is a chronic non-specific inflammatory disease that invades colonic mucosa without gender advantage (Matsuoka et al., 2018). The peak age of IBD is between 20 and 40 years old (Loftus, 2004; Bernstein et al., 2006; Cosnes et al., 2011). The main symptoms of IBD are abdominal pain, diarrhea, mucous bloody stool, as well as extra-intestinal symptoms. IBD is mostly common in developed countries including North America, Europe, Australia, and New Zealand, with incidence rate as high as 20–100 per million people. It is estimated that as many as 1 million Americans suffer from IBD (Cohen et al., 2010; Magro et al., 2012). In recent decades, the incidence of IBD has been rising all over the world, especially in East Asian (Loftus et al., 2007; Bengtson et al., 2009; Cosnes et al., 2011; Molodecky et al., 2012).

The pathogenesis of IBD has not been fully elucidated. It has a strong genetic determinant. For instance, first-degree relatives of patients with IBD are 4 to 20 times more likely to develop IBD (Kevans et al., 2016). Recent genome-wide association studies (GWASs) have identified more than 200 responsible genomic loci associated with IBD (Turpin et al., 2018). Despite these fruitful findings, its pathogenic mechanism has not been fully understood yet. On the other hand, gut microbiota may be related to the pathogenesis of IBD (Nishida et al., 2018). Imbalance of gut microbiota coupled with impaired intestinal bacterial clearance could enhance the invasiveness of pathogens, disrupt intestinal immune response, accelerate intestinal inflammation, and eventually lead to IBD. In a recent controlled trial, patients in the fecal microbiota transplantation group showed significant clinical improvement, indicating that high-dose fecal microbiota transplantation is an effective method for the treatment of active IBD (Paramsothy et al., 2017). Another study indicates that the low abundance of *Phascolarctobacterium* is positively correlated with the occurrence of IBD (Bajer et al., 2017).

Although previous extensive studies have established observational associations between gut microbiota and IBD developing risk, the causal nature is largely unclear. Mendelian randomization (MR) analysis is a statistical approach that aims to infer causal relationship from observational association results (Lee and Lim, 2019). With the rapidly increasing genetic data at both microbiota and complex disease sides, MR has been widely applied in recent years. MR approach has three essential assumptions: (1) Instrumental variable (IV) is strongly associated with exposure; (2) IV is not associated with any confounders of exposure; and (3) The association of IV with outcome is only through exposure. It has been used to infer the causal relationship from gut microbiota to type 2 diabetes, neurodegenerative diseases, and bone density (Burgess et al., 2013; Bowden et al., 2015; Goodrich et al., 2016; Quigley, 2017; Verbanck et al., 2018).

In the present study, in order to explore the causal relationship from gut microbiota to IBD, and to identify specific pathogenic bacteria taxa, we conducted a two-sample MR study based on GWAS summary data. In brief, summary data from the microbiota GWAS (MGWAS) of the TwinsUK study were used as exposure, and GWAS summary statistics from two IBD GWAS were used as discovery and replication outcomes.

## Materials and Methods

### GWAS Summary Statistics

The MR analysis was performed on GWAS summary statistics of both microbiota and IBD. All data were retrieved from previously published studies that were released to the public.

The microbiota GWAS summary statistics from the TwinsUK study (Goodrich et al., 2016) served as exposure. In brief, The TwinsUK study collected 3,261 fecal samples from 1,126 twin pairs from the TwinsUK Registry in British. Microbiota 16S rRNA was sequenced using Illumina Miseq 2 × 250 bp sequencing platform, followed by host genome genotyping using Illumina HumanHap610 Quad Chip. For genotype imputation, the 1,000 Genomes project (Phase 3) reference panel was used. Sixty-one bacteria taxa were found to be associated with 307 host SNPs with *p*-values ranging from 7.33 × 10^–5^ to 4.94 × 10^–9^ (Supplementary Table 1).

The discovery outcome sample UK IBD and Understanding Social Program (UKIBD and USP) is a GWAS study based on a general prospective population cohort of European ancestry with 12,924 cases and 35,391 controls. Host genome was genotyped by the HumanCyto SNP-12 BeadChip and the Immunochip arrays, and was imputed into the UK IBD Genetics Consortium and UK10K Consortium reference panel (Burgess et al., 2013). A total of 38 genomic loci were identified at the genome-wide significance level (*p* < 5.0 × 10^–8^), increasing the number of known IBD risk sites to 200.

The replication British IBD sample was the GWAS of 16,452 IBD British cases and 18,837 controls. Participants were genotyped on the Human Core Exome v12.1, the Affymetrix 500K, or the Affymetrix 6.0 genotyping array.

### Instrumental Variable Selection

The same criteria were used for IV selection in both discovery and replication samples. IVs were grouped at family or genus level. Specifically, a bacterial feature was defined as a family or genus. SNPs associated with bacterial taxa in one feature were grouped together for that feature. As a QC step, palindrome SNPs, which are defined as SNPs with ambiguous strand information (e.g., A/T or G/C polymorphisms), were removed. SNP-feature association threshold was set to be 1.0 × 10^–5^. In order to eliminate the effect of linkage disequilibrium (LD), SNPs within each feature were clumped with PLINK (v1.9). The LD threshold was set to be *r*^2^ < 0.1, and the clustering window was set to be 500 kb. LD was estimated on the 1,000 Genome Project sequencing data (Phase 3).

In order to minimize the effect of horizontal pleiotropy. MR-PRESSO global test and outlier test were applied (Verbanck et al., 2018). The MR-PRESSO outlier test calculates the *p*-value for the significance of pleiotropy for each SNP, while the MR-PRESSO Global test calculates the *p*-value for the overall level of pleiotropy. Each individual SNP was deleted in turn and the MR-PRESSO Outlier test was applied to the set of remaining SNPs (Verbanck et al., 2018). All significant SNPs were removed. A MR-PRESSO Global test was finally performed to monitor the overall pleiotropic effect. Non-significant SNPs were used for subsequent MR analysis.

### MR Analysis

Upon the selection of qualified SNPs, MR analysis was then performed for a causal relationship from microbiota feature to IBD risk. Specifically, each microbiota feature was tested for its association. For features with multiple IVs, the inverse-variance weighted (IVW) test (Burgess et al., 2013) was applied. For features with only one IV, the Wald ratio test was applied. The results of IVW were also cross-validated by three alternative tests, including the MR-Egger regression (Bowden et al., 2015), the weighted median estimator (Bowden et al., 2016) and the MR-PRESSO (Verbanck et al., 2018).

Nominally significant results identified in the discovery sample were subjected to be replicated in the replication sample, with the same analysis procedures.

The horizontal heterogeneity effect was examined by the IVW test and the MR-Egger regression. Meanwhile, a leave-one-out sensitivity analysis was performed to monitor if significant associations were dominated by a single SNP.

All the above analyses (including sensitivity analysis and MR analysis) were implemented within the R packages TwoSampleMR^1^ (Hemani et al., 2018) and MRPRESSO^2^ (Sanna et al., 2019).

## Results

**FIGURE 1:**
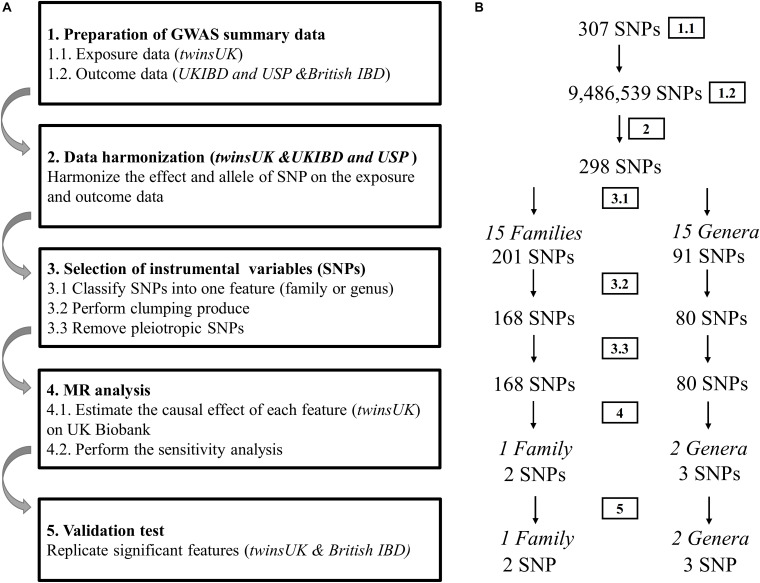
Diagrammatic description of MR analysis in the discovery and replication. **(A)** The whole workflow of MR analysis. **(B)** The main results and the change in the number of SNPs.

The flow chart of the present study is displayed in Figure 1. In the discovery sample, there are 237 host SNPs that are associated with gut microbiota features at the significance threshold *p* < 1.0 × 10^–5^. After clumping, 168 and 80 SNPs are left for 15 families and 15 genera, respectively (Supplementary Table 2). Two families with the largest number of SNPs are *Lachnospiraceae* (51 SNPs) and *Ruminococcaceae* (51 SNPs), followed by *Bacteroidaceae* (36 SNPs). There are five families, *Barnesiellaceae*, *Enterobacteriaceae*, *Rikenellaceae*, *Streptococcaceae*, and *Veillonellaceae*, each having only one SNP. At the genus level, the genus with the largest number of SNPs is *Bacteroides* (36 SNPs), followed by *Faecalibacterium* (9 SNPs) and *Coprococcus* (6 SNPs). There are four genera each having only one SNP, *Anaerostipes*, *Dorea*, *Streptococcus*, and *Veillonella*. Of note, genus is a child taxon of family, therefore SNPs contained in both features may overlap. For example, the genus *Faecalibacterium* is a child taxon of the family *Ruminococcaceae*. The SNPs in them are partly identical.

For features containing multiple IVs, no outliers were detected using the MR-PRESSO outlier test and no evidence of horizontal pleiotropy (both MR-PRESSO Global test *p* > 0.05/15 = *p* > 3.3 × 10^–3^ and MR-Egger regression *p* > 0.05) was observed.

### MR Analysis

In the discovery sample, after removing potentially pleiotropic SNPs, one family and two genera are significant at the nominal level (*p* < = 0.05): family *Verrucomicrobiaceae* (2 IVs, beta = 0.04, *p* = 0.05), genus *Akkermansia* (2 IVs, beta = 0.04, *p* = 0.05) and genus *Dorea* (1 IV, beta = −0.07, *p* = 0.04).

In sum, three features (one family ++ two genera) are causally associated with IBD in the discovery sample. These three features were replicated in the British IBD replication sample. The same IVs are available in the replication sample. Using the same IVW test, the replication *p*-value is significant (*p* = 0.02) and the effect direction is consistent for family *Verrucomicrobiaceae* and genus *Akkermansia* (Table 1). For the other genus *Dorea*, only one SNP rs10743315 is qualified as the IV. Using the Wald ratio test, the MR *p*-value is 0.01, again with consistent effect direction. Moreover, there is no evidence of heterogeneity at the three identified features in both discovery and replication sample. Detailed information of the 3 IVs is listed in Table 2.

**TABLE 1:** MR analysis of gut microbiota on IBD in both discovery and replication samples.

**Stage**	**MR Tests**	**Family**			**Genus**					
		***Verrucomicrobiaceae***			***Akkermansia***			***Dorea***		
		**No. SNP**	**b_xy_**	***p*-value**	**No. SNP**	**b_xy_**	***p*-value**	**No. SNP**	**b_xy_**	***p*-value**
**Discovery**										
	IVW	2	0.04	**0.05**	2	0.04	**0.05**	–	–	–
	Wald ratio test	–	–	–	–	–	–	1	−0.07	**0.04**
**Replication**										
	IVW	2	0.02	**0.02**	2	0.02	**0.02**	–	–	–
	Wald ratio test	–	–	–	–	–	–	1	−0.08	**0.01**

*No. SNP is the number of SNPs being used as IVs. b_*xy*_ is the estimated effect coefficient. Significant *p*-values were marked in bold. IVW, inverse-variance weighted.*

**TABLE 2:** Instrumental variables used in both discovery and replication studies.

**Stage**	**Snp**	**Chr**	**Position**	**Locus**	**A1**	**A0**	**Closest Gene**	**Exposure**			**Outcome (Discovery)**			**Outcome (Replication)**		
								**Beta**	**SE**	***p*-value**	**Beta**	**SE**	***p*-value**	**Beta**	**SE**	***p*-value**
Family *Verrucomicrobiaceae*/	rs10081087	6	141858751	6q24.1	A	G	RN7SKP106	0.57	0.12	2.16 × 10^–7^	0.04	0.02	0.045	0.04	0.02	0.05
Genus Akkermansia	rs692899	18	43316270	18q12.3	C	T	SLC14A1	−0.55	0.11	1.83 × 10^–8^	−0.01	0.02	0.42	−0.02	0.02	0.16
Genus *Dorea*	rs12607607	18	39082090	18q12.3	T	C	KC6	−0.63	0.11	3.69 × 10^–9^	0.04	0.02	0.04	0.05	0.02	0.01

*Chr is Chromosome of SNP. Physical position is based on the human genome GRCH37 assembly. A1 is the effect allele and A0 is the other allele. Beta is the estimate coefficient of the effect allele. SE is the standard error of the estimate coefficient. Closest gene is the closest gene to which the SNP is mapped. Family *Verrucomicrobiaceae* and genus *Akkermansia* have the same SNPs.*

## Discussion

In this study, we used MR analysis to evaluate the causal relationship between gut microbiota and IBD. Using the summary statistics of one microbiota GWAS and 2 IBD GWASs, we identified and replicated three bacterial taxa, one family *Verrucomicrobiaceae* and two genera *Akkermansia* and *Dorea*, that may have causal relationship with IBD. Our study confirmed that gut microbiota can aggravate IBD, suggesting that gut microbiota plays a regulatory role in IBD.

The gut microbiota is an intricate and dynamic collective of ecological microbial communities that are colonized in the human gut, even called a “forgotten organ” (O’Hara and Shanahan, 2006; Backhed et al., 2015). Gut microbiota is not only an important part of immune and metabolic health, but also regulate central nervous system and relevant disorders, including movement disorders, neurodegenerative diseases, behavioral disorders, neuroimmune-mediated diseases, and Cerebrovascular accident (Strandwitz, 2018). More than 90% of the gut microbiota that maintain intestinal health and balance in adults consist of four phylums of *Firmicutes*, *Bacteroides*, *Actinobacteria*, and *Proteobacteria* (Matsuoka and Kanai, 2015). The large intestine comprises the densest and metabolism-active microorganism in healthy individuals, which is predominated by anaerobic microbiota, two phyla *Firmicutes* and *Bacteroidetes*, apart from *Actinobacteria*, *Proteobacteria*, and *Verrucomicrobia* (Eckburg et al., 2005).

The *Dorea* identified in this study belongs to the *Lachnospiraceae* family, which mainly exists in the gut microbiota of mammals and humans. One previous study has established a link between *Lachnospiraceae* and IBD (Lee et al., 2020). Another recent studies has also confirmed that the level of *Lachnospiraceae* and butyric acid gets decreased in IBD patients (Sasaki et al., 2019). The genus *Akkermansia* is present abundantly in the human gastrointestinal tract where it is believed to be a key symbiont member of the microbiota (Collado et al., 2007; Derrien et al., 2008; van Passel et al., 2011; Clarke et al., 2014; Guo et al., 2016). Extensive studies demonstrate that the lower level of *Akkermansia* is found in patients with IBD and other metabolic disorders, suggesting that *Akkermansia* may have potential anti-inflammatory properties (Zhang et al., 2016).

Previous studies have shown that the imbalance of gut microbiota is one of the pathogenic factors of IBD, but the specific regulatory mechanism is yet poorly understood. One possible mechanism, among others, is that the anti-inflammatory activity of IBD model is related to the regulation of inflammatory cytokines such as iNOS, MPO, IL-4, IL-10, EGF, MUC2, IL-6 and so on (Ma et al., 2018). However, this needs to be confirmed by further functional studies, which is beyond the scope of this study.

Mendelian randomization analysis is an effective method to explore causality from exposure to outcome while controlling confounding factors. The MR analysis in this study has the following advantages. First, it is a new attempt to speculate the causal relationship from gut microbiota to IBD, which provides a theoretical basis for the follow-up study of the regulation mechanism of single strain on IBD. Second, it is based on publicly available large-scale GWAS summary statistics, so it provides an effective choice for mining reliable genetic information without additional experimental cost.

Obviously, our study has certain limitations. First, due to limited sample size, the genetic loci identified in gut microbiota GWAS are still limited, which limits the statistical power of MR analysis. Second, MR analysis based on one single IV is less robust, which may bias the interpretation of our findings.

In conclusion, we evaluated the causal relationship from gut microbiota to IBD and identified specific bacterial taxa that may affect the pathogenesis of IBD by a two-sample MR analysis using publicly available GWAS summary statistics. Our results provide a basis for revealing the causal relationship from gut microbiota to IBD, and thus offer new insights into its development and treatment.

## Data Availability Statement

Publicly available datasets were analyzed in this study. This data can be found here: ftp://ftp.sanger.ac.uk/pub/project/humgen/summary_statistics/human/2016-11-07/. The accession code EGAS00001000924. Key data were supplied in the Supplementary Tables 1, 2.

## Ethics Statement

Ethical review and approval was not required for the study on human participants in accordance with the Local Legislation and Institutional Requirements. The patients/participants provided their written informed consent to participate in this study. Written informed consent was obtained from the individual(s) for the publication of any potentially identifiable images or data included in this article.

## Author Contributions

Z-JZ: conceptualization, formal analysis, data curation, and writing – original draft. H-LQ: validation and writing – original draft. NZ: formal analysis and writing – original draft. JW: resources and writing – original draft. X-YW: data curation and writing – original draft. BL: data curation, supervision, methodology, writing, and revising. RH: conceptualization, methodology, software, data curation, writing, review, editing, and supervision. All authors contributed to the article and approved the submitted version.

## Conflict of Interest

The authors declare that the research was conducted in the absence of any commercial or financial relationships that could be construed as a potential conflict of interest.

## Footnotes

1 https://github.com/MRCIEU/TwoSampleMR

2 https://github.com/rondolab/MR-PRESSO

## Abbreviations

FDR: false discovery rate
GWAS: genome-wide association study
IBD: inflammatory bowel disease
IV: instrumental variable
IVW: inverse-variance weighted
LD: linkage disequilibrium
MGWAS: microbiome genome-wide association study
MR: Mendelian randomization
OTU: operational taxonomic unit
UKB: UK Biobank
USP: understanding social program.
